# Respiratory outcomes after cleft palate closure in Robin sequence: a retrospective study

**DOI:** 10.1007/s00784-024-05647-w

**Published:** 2024-04-11

**Authors:** Nathaniel A. T. Sullivan, Nadia Lachkar, J. Peter W. Don Griot, Frea H. Kruisinga, Wendela G. Leeuwenburgh-Pronk, Chantal J. M. Broers, Corstiaan C. Breugem

**Affiliations:** 1 Amsterdam UMC, Department of Plastic Surgery, Location University of Amsterdam, Emma Children’s Hospital, Meibergdreef 9, Amsterdam, The Netherlands; 2 Amsterdam Reproduction and Development Research Institute, Amsterdam, The Netherlands; 3 Department of Pediatrics, Amsterdam UMC, Location University of Amsterdam, Emma Children’s Hospital, Meibergdreef 9, Amsterdam, The Netherlands

**Keywords:** Robin sequence, Cleft palate, Risk factors, Respiratory distress

## Abstract

**Objectives:**

There is a paucity of information about the possible risk factors that could identify patients with Robin sequence (RS) who are more prone to developing obstructive airway complications after palate closure. This study aimed to compare the respiratory complication rates in patients with RS and isolated cleft palate (ICP).

**Materials and methods:**

In this retrospective study, we reviewed the medical records of 243 consecutive patients with RS and ICP who were treated at Amsterdam University Medical Centers over the past 25 years. We collected preoperative data on previous treatment, diagnostic findings, surgical technique, weight, and presence of congenital anomalies.

**Results:**

During cleft palate closure, patients with RS were older (11.9 versus 10.1 months; *p* = 0.001) and had a lower gestational age than those with ICP (37.7 versus 38.5 weeks; *p* = 0.002). Patients with RS had more respiratory complications (17 versus 5%; *p* = 0.005), were more often non-electively admitted to the pediatric intensive care unit (PICU) (13 versus 4.1%; *p* = 0.022), and had a longer hospital stay duration (3.7 versus 2.7 days; *p* = 0.011) than those with ICP. The identified risk factors for respiratory problems were a history of tongue-lip-adhesion (TLA) (*p* = 0.007) and a preoperative weight of < 8 kg (*p* = 0.015). Similar risk factors were identified for PICU admission (*p* = 0.015 and 0.004, respectively).

**Conclusions:**

The possible risk factors for these outcomes were a low preoperative weight and history of TLA. Closer postoperative surveillance should be considered for patients with these risk factors.

**Clinical relevance:**

Identifying risk factors for respiratory complications could provide clinicians better insight into their patients and allows them to provide optimal care for their patients.

## Introduction

Robin sequence (RS) is a triad of micrognathia, glossoptosis, and concomitant upper airway obstruction (UAO) [1]. Although not required for a diagnosis, a wide U-shaped cleft palate is present in 90% of RS cases [1]. First described in 1923 by French stomatologist Pierre Robin [2], RS has an incidence of 1 in 8,000–14,000 newborns [3–5], and is often associated with certain syndromes or other anomalies. However, it may also be present as an isolated entity without other anomalies [6–9].

Patients with RS present with varying degrees of UAO and feeding problems, which in turn can lead to life-threatening respiratory and cardiac sequelae and failure to thrive if not adequately addressed [10, 11]. The presence of a cleft palate makes providing care more complex and influences comorbidities. Mortality rates of 1–26% have been reported, often in patients with RS and associated malformations [10, 12].

Following palatoplasty, all patients are monitored for possible bleeding and respiratory distress [13]. This is especially of concern in some syndromic patients, including those with RS, as they are already at risk of airway obstruction because of tongue retrodisplacement [14, 15].

There are unique challenges and considerations in the treatment of cleft palate in patients with RS. Questions remain about adverse surgical outcomes after palatoplasty compared to isolated cleft palate (ICP) [16–18]. In particular, the literature is scarce regarding whether the presence of RS negatively impacts postoperative respiratory problems for palatoplasty. The current literature suggests that patients with RS have more obstructive respiratory problems following palatoplasty. Respiratory outcomes are defined differently and treatment protocols vary widely among articles [19–21]. Little is also known about the pediatric intensive care unit (PICU) admission rates. Identifying at-risk patients is imperative for achieving optimal care in pediatric patients [22]. Therefore, identifying the risk factors can greatly improve surgical care.

Current practice patterns for the diagnosis and management of UAO in patients with RS varies geographically [23, 24]. There are several different treatment protocols for managing UAO in RS and it is unknown whether these protocols affect postoperative outcomes after palatoplasty.^19^ Our treatment protocol has been previously described [25, 26].

Objective assessment of UAO in patients with RS is important to adequately adjust management for optimal care. Polysomnography (PSG) is a powerful tool to objectively assess the degree of UAO in patients with RS [27]. Two studies described methods to assess the feasibility of palatoplasty in patients with RS. Although airway compromise was not uniformly defined, both studies relied on PSG to assess patients’ eligibility for palatoplasty [19, 21].

This study aimed to compare the respiratory complication rates in patients with RS and ICP. Furthermore, we aimed to describe the risk factors associated with adverse respiratory complications, hospital stay duration, and the PICU admission rate. We hypothesized that patients with RS would develop more airway problems than those with ICP. The goal of this study is to add new evidence-based literature on respiratory problems after palatoplasty in patients with RS. We believe that identifying the risk factors for palatoplasty could aid clinicians in their decision-making regarding when to operate and how to approach postoperative care. Identifying these risk factors could improve quality of care and possibly improve surgical outcomes.

## Materials and methods

This review was conducted following the Strengthening the Reporting of Observational Studies in Epidemiology (STROBE) guidelines.

### Ethics statements

#### Ethical approval

for this study was obtained from the Human Research Ethics Committee of the Amsterdam University Medical Centers (AUMC) (W22_320 # 22.407). Informed consent was obtained from all individual patients included in the study.

### Study design and population

In this retrospective, single-center, observational controlled cohort study, we examined the postoperative outcomes after primary palatoplasty in patients with RS. Our study group consisted of patients with RS and a cleft palate, either isolated, with a syndromic diagnosis or without a syndromic diagnosis but with congenital anomalies present. The control group comprised of patients with ICP and no associated congenital malformations. All included patients were treated at AUMC in the previous 25 years (January of 1997 to August 2022). Patients without perioperative palatoplasty data were excluded. All eligible participants received a letter containing information about the research project.

### Data acquisition

All data were captured and stored using Castor Electronic Data Capture.

Isolated RS refers to cleft palate and RS in the absence of other anomalies. Syndromic RS refers to cleft palate and RS with a diagnosis of a syndrome or RS in the absence of a diagnosis of a syndrome but with congenital anomalies (often referred to as RS-plus). The primary outcome was postoperative respiratory- or feeding-related complications during the initial postoperative hospitalization after palatal closure. Respiratory complications were defined as any objective obstructive airway problems that required PICU admission or subsequent treatment (positional change, post-surgical reintubation, nasopharygeal airway placement, high-flow nasal cannula, or tracheostomy). Feeding-related complications were defined as inadequate oral intake that postponed discharge. A stable oral intake is a prerequisite for the surgery. The variables gathered included gestational age, birth weight and length, the presence of cardiac congenital anomalies or congenital anomalies regarding the central nervous system (neurological), diagnosis of a syndrome, prior treatments, prior polysomnography (PSG) results from the first week of life before airway interventions, preoperative weight and length during palatoplasty, surgical technique, length of hospital stay, and PICU admission rate and length of stay. Only the patients who required emergency PICU admission (and not those who were planned) were included in this subgroup.

### Statistical analysis

Normality was tested using the Shapiro–Wilk test. The Mann–Whitney-U test was used to determine differences of the means for continuous variables and the Pearson *X*^2^ test or Fisher exact test was used to compare categorical data between the groups. Univariate binary logistic regression analysis was used to identify variables associated with the outcomes and multivariate binary logistic regression analysis was used to correct for confounders. Differences were considered statistically significant at *p* < 0.05. Statistical analyses were performed using SPSS (version 28; IBM Corp., Armonk, NY, USA).

## Results

### Demographics

Between September 2022 and November 2022, we enrolled 243 consecutive patients: 100 with ICP and 143 with RS. Fifty-five patients in the RS group had syndromic RS and 88 had isolated RS.

The patient demographics are presented in Table 1. Patients with RS were older at time of cleft palate repair (11.9 ± 15.3 versus 10.1 ± 4.2 months; *p* = 0.001) and had a lower gestational age than those with ICP (37.7 ± 2.8 versus 38.5 ± 3.1 weeks; *p* = 0.002). Patients with syndromic RS had a lower gestational age (37.0 ± 2.7 versus 38.2 ± 2.9 weeks; *p* = 0.002), lower birth weight (2.7 ± 0.8 versus 3.3 ± 0.8; *p* = 0.001), and lower preoperative weight than those with isolated RS (7.6 ± 1.1 versus 8.9 ± 1.3 kg; *p* = 0.004).

**Table 1:** Comparison of the ICP and RS groups and comparison of subgroups isolated RS and syndromic RS

	ICP group (*n* = 100)	RS group (*n* = 143)	p	Syndromic RS (*n* = 55)	Isolated RS (*n* = 88)	p
Presence of syndrome	0/100 (0%)	55/143 (38%)	-	-	-	-
Preoperative PSG	0/100 (0%)	29/143 (20%)	-	13/55 (24%)	16/88 (18%)	0.417
Gestational age (weeks)	38.5 (± 3.1)	37.7 (± 2.8)	0.002*	37.0 (± 2.7)	38.2 (± 2.9)	0.002*
Weight at birth (kg)	3.2 (± 0.6)	3.0 (± 0.8)	0.140	2.7 (± 0.8)	3.3 (± 0.8)	0.001*
Mean age at the time of cleft palate repair (months)	10.1 (± 4.2)	11.9 (± 15.3)	0.001*	11.1 (± 12.0)	10.8 (± 2.7)	0.206
Weight during cleft palate repair (kg)	8.8 (± 1.5)	8.4 (± 1.4)	0.269	7.6 (± 1.1)	8.9 (± 1.3)	0.004*
Respiratory complications §	5/100 (5%)	25/143 (17%)	0.005*	12/55 (22%)	13/88 (15%)	0.295
Positional change only	1	1	0.310	0	1	0.520
Antibiotics	1	3	0.520	1	2	0.496
NPA	0	1	0.588	1	0	0.387
Post-surgical reintubation	3	13	0.049*	6	6	0.250
HFNC	0	9	0.008*	5	4	0.234
Tracheostomy	0	1	0.588	1	0	0.387
Feeding-related complications	11/100 (11%)	9/143 (6.3%)	0.237	6/55 (11%)	3/88 (3.4%)	0.089
Non-elective postoperative Intensive care admission †	4/98 (4.1%)	18/137 (13%)	0.022*	10/53 (19%)	8/83 (10%)	0.121
Average length of intensive care stay (days)	1.5 (± 0.6)	2.4 (± 1.9)	0.696	2.3 (± 2.1)	2.4 (± 1.8)	0.917
Length of hospital stay (days)	2.7 (± 1.6)	3.7 (± 4.4)	0.011*	4.7 (± 6.6)	3.0 (± 1.7)	0.375

*Significant differences in results

ICP = isolated cleft palate, RS = Robin sequence, PSG = polysomnography, NPA = nasopharyngeal airway, HFNC = high-flow nasal cannula

† Not all parameters are known for all patients, due to missing data total number of patients does not match

§ Some patients required multiple interventions, therefore the sum does not add up to total respiratory complications

### Syndromes

Patients with syndromic RS had 38 different syndromes: Stickler syndrome (*n* = 20), Cornelia de Lange syndrome (*n* = 3), 22q11-deletion syndrome (*n* = 3), Nager syndrome (*n* = 2), and Moebius syndrome (*n* = 2) were the most commonly occurring syndromes. Thirteen patients had a cleft palate with congenital anomalies but no syndromic diagnosis.

### Complications

Respiratory complications occurred in 25 (18%) patients with RS compared to 5% (*n* = 5) of patients with ICP (*p* = 0.005). Post-surgical reintubation (9.1%, *n* = 13 vs. 3.0%, *n* = 3) and HFNC (6.3%, *n* = 9 vs. 0%, *n* = 0) occurred more frequently in patients with RS compared to patients with ICP (*p* = 0.049 and *p* = 0.008). There were no significant differences between the syndromic and isolated RS groups (*p* = 0.295). No differences were observed in feeding-related complications between the RS and ICP groups (*p* = 0.237), or between the syndromic RS and isolated RS groups (*p* = 0.089) (Table 1).

Patients with RS who had previously undergone tongue-lip-adhesion (TLA) had more respiratory complications after cleft palate surgery (40%; *n* = 12; *p* = 0.024). No other previous primary treatment methods resulted in an increase in respiratory complications after the palatoplasty. No increase in feeding-related complications was observed in any of the previous treatment modalities (Table 2).

**Table 2:** Primary management of UAO in RS patients and its statistical correlation with airway-related and feeding-related complications after palatoplasty compared to the overall average in RS group

Management	Usage†		Airway-related complications			Feeding-related complications		
	%	n	%	n	p	%	n	p
Prone/side positioning	27	38	24	9	0.622	8	3	1.000
HFNC	6.9	10	20	2	1.000	20	2	0.147
CPAP	6.2	9	22	2	1.000	22	2	0.128
Intubation	4.9	7	0	0	0.600	14	1	0.353
NPA	14	20	15	3	0.761	0	0	0.343
Glossopexy	20	30	40	12	0.024*	10	3	0.701
MDO	2.2	3	0	0	1.000	0	0	1.000
Tracheostomy	0.8	2	0	0	1.000	0	0	1.000

*Significant differences in results

RS = Robin sequence, HFNC = high flow nasal cannula, CPAP = continuous positive airway pressure, NPA = nasopharyngeal airway, MDO = mandibular distraction osteogenesis

†No data on prior treatment was available for some patients, and some patients had multiple treatments, therefore percentages do not add up to 100%

No differences were observed in either airway-or feeding-related complications between the surgical techniques used for palate closure (Table 3).

**Table 3:** Surgical techniques for palatoplasty and its effect on airway-related and feeding-related complications

Surgical technique	Usage		Airway-related complications			Feeding-related complications		
	%	n	%	n	p	%	n	p
von Langenbeck palatoplasty	77	188	12	22	0.641	10	19	0.052
Veau-Wardill-Killner VY-pushback	14	35	14	5	0.781	0.0	0	0.088
Furlow double opposing Z-plasty	3	7	14	1	1.000	0.0	0	1.000

### Congenital anomalies

Nineteen patients (34%) in the syndromic RS group had ≥1 cardiovascular anomaly. Thirteen (24%) patients had congenital neurological anomalies.

### Hospital stay

Patients with RS had a longer hospital admission than those with ICP (3.7 days ± 4.4 versus 2.7 ± 1.6 days; *p* = 0.011). There were no differences in hospital stay between patients with syndromic RS and those with isolated RS (4.7 ± 6.6 versus 3.0 ± 1.7 days; *p* = 0.375) (Table 1).

Nine patients with RS were electively admitted to the PICU, all these patients had a history of TLA. Patients with RS were more often non-electively admitted to the PICU after palatoplasty than those with ICP (13%, *n* = 18 versus 4.1%, *n* = 4; *p* = 0.022). There were no differences between the length of PICU stay between the RS and ICP groups (2.4 ± 1.9 versus 1.5 ± 0.6 days; *p* = 0.696), and there were there no differences PICU duration of stay patients with syndromic RS and those with isolated RS (2.3 ± 2.1 days versus 2.4 ± 1.8 days; *p* = 0.917) (Table 1).

### Logistic regression analysis

The results of multivariate logistic regression analysis are presented in Table 4. A history of TLA and preoperative weight of < 8 kg were associated with a four-fold (odds ratio [OR]: 3.975; 95% confidence interval [CI]: 1.458–10.839; *p* = 0.007) and five-fold increased risk of developing respiratory complications, respectively (OR: 4.760; 95% CI: 1.357–16.694; *p* = 0.015). Furthermore, TLA (OR: 4.025; 95% CI: 1.306–12.395; *p* = 0.015) and preoperative weight of < 8 kg (OR: 10.358; 95% CI: 2.104–51.001; *p* = 0.004) were associated with PICU admission. PSG was performed in only 29 patients with RS during the first month of life. A higher apnea-hypopnea index was associated with PICU admission after palatoplasty (OR: 1.072; 95% CI: 1.008–1.140; *p* = 0.027).

**Table 4:** Logistic regression analysis for variables associated with RS to predict surgical outcomes

	Respiratory complications			ICU admission		
Variable	OR	95% CI	p	OR	95% CI	p
Age during cleft palate repair	1.007	0.980–1.035	0.605	1.006	0.991–1.020	0.438
Male	0.939	0.444–1.988	0.870	1.554	0.645–3.747	0.326
*Diagnosis*						
ICP	Ref			Ref		
RS	4.025	1.485–10.915	0.006*	3.555	1.164–10.858	0.026*
Isolated RS	Ref			Ref		
Syndromic RS	1.589	0.666–3.792	0.297	2.180	0.800-5.941	0.128
*Airway intervention*						
Prone/side positioning	1.313	0.498–3.463	0.582	0.549	0.161–1.867	0.337
HFNC	0.989	0.200–4.900	0.989	1.533	0.300–7.850	0.608
CPAP	1.099	0.219–5.517	0.909	1.704	0.328–8.836	0.526
NPA	0.593	0.186–2.614	0.593	1.191	0.303–4.691	0.802
Glossopexy	3.975	1.458–10.839	0.007*	4.024	1.306–12.395	0.015*
*PSG*						
AHI	1.048	0.998–1.101	0.062	1.072	1.008–1.140	0.027*
ODI	1.015	0.954–1.079	0.645	1.027	0.949–1.101	0.444
Average saturation	1.095	0.600-1.999	0.767	0.990	0.526–1.863	0.974
Lowest saturation	0.980	0.889–1.080	0.685	0.966	0.875–1.067	0.497
*Congenital anomalies*						
Cardiovascular	2.692	0.601–12.056	0.305	1.015	0.199–5.188	0.986
Neurological	1.613	0.333–7.815	0.553	0.998	0.165–6.051	0.999
Preoperative weight (continuous)	0.646	0.426–0.980	0.040*	0.380	0.191–0.754	0.006*
Preoperative weight (< 8 kg)	4.760	1.357–16.694	0.015*	10.358	2.104–51.001	0.004*

Ref = reference value OR = Odds ration; CI = confidence interval; ICP = isolated cleft palate; RS = Robin sequence; HFNC = high flow nasal cannula; CPAP = continuous positive airway pressure; NPA = nasopharyngeal airway; AHI = apnea-hypopnea index; ODI = oxygen desaturation index

## Discussion

We have demonstrated that respiratory complications after palatoplasty occur more often in patients with RS than in those with ICP, which supports our hypothesis. We also found that hospital admissions increased and that PICU admissions were more frequent in patients with RS than in those with ICP. Furthermore, we identified risk factors, such as previous TLA and low preoperative weight, which are associated with worse postoperative outcomes.

Respiratory complications after palatoplasty occurred in 25 patients (17,5%) with RS and in 5 patients (5%) with ICP. These findings are consistent with results of previous studies that identified a higher incidence of respiratory problems after palatoplasty in patients with RS than in those with ICP. Prior studies have provided heterogeneity for this outcome measure (0–47.4% in RS and 2.7–8% in ICP), possibly due to varying definitions of respiratory problems being included [19–21, 28–31]. Costa et al. and van Lieshout et al. reported respiratory complications in 6% and 30%, respectively [19, 21]. The first study defined respiratory complications as those requiring readmission, whereas the other did not provide a definition. Other studies have described different definitions of respiratory complications, such as lingual edema, intubation difficult, and delayed extubation [16, 20, 28–31]. The lack of consensus in defining respiratory complications makes it difficult to compare studies. Two studies used PSG to determine the eligibility for palatoplasty [19, 21]. In our patient group, historically, no preoperative PSGs were routinely performed to assess eligibility for surgery; however, the prevalence of respiratory complications and PICU admission was similar to that in other studies who performed PSG routinely [19, 21]. These differences can be explained by the small sample size or the use of different definitions of respiratory problems. However, future studies are needed to investigate the advantages and disadvantages of perioperative PSG, and they should look at the impact of preoperative PSG could have on timing of cleft palate surgery in RS patients.

It has been suggested that patients with RS develop more respiratory complications after palatoplasty than their ICP counterparts because of their smaller oropharynx after palatoplasty [21, 32]. Therefore, it is reasonable to assume that an increased luminal size of the oropharynx after mandibular distraction osteogenesis (MDO) could improve respiratory outcomes [33]. We only had 2 patients with MDO, and both did not developed respiratory problems after palatoplasty. This compares to the study of Costa et al. [19], where patients after MDO did not significantly have more breathing problems after palatoplasty. Moreover, our results demonstrated that prior TLA is associated with an increased risk of developing postoperative respiratory complications after palatoplasty. This can possibly be explained by the division of the tongue-lip adhesion, which is often (in 85% of cases in our cohort) performed during palatoplasty, leading to dorsal placement of the base of the tongue in some patients [34]. This suggests that the optimal timing for dividing the TLA should not coincide with cleft palate surgery, but rather later when post-operative swelling has significantly subsided. It also highlights a need for more thorough pre-operative assessments in RS patients, particularly for those who underwent TLA, to determine their suitability for cleft palate repair. Employing objective preoperative evaluation techniques, such as PSG and blood gas analysis could assist clinicians in ensuring that a patient is adequately prepared and fit for the surgical procedure. Another possible explanation is that TLA is performed in the most severe cases of UAO in RS and does not increase the volume of the oropharynx since no mandibular lengthening us performed [35]. However, this study was not performed looking at treatment indications for breathing problems after birth, but definitely brings up the discussion of the impact that primary treatment of obstructive breathing after birth could have on cleft palate surgery. Furthermore, it suggests the need to better evaluate RS patients pre-operatively. We found no significant differences in feeding and respiratory complications between the surgical techniques. These results reflect on those of Van Lieshout et al. who also found the same results [21]. As there are no differences in outcomes, it is advised to choose a surgical technique based on other variables, such as the chance of the patient developing oronasal fistulae, speech outcomes, and the surgeons’ preference [36–39].

The lengths of hospital stay and PICU admission are both important outcome measures for quality of care [40]. Moreover, pediatric PICU admission is associated with long-term psychological effects on both patients and parents [41, 42]. The length of hospital stay was longer in the RS group than in the ICP group. These results are comparable to those reported by Costa et al. who found the same result, although it was not significant. The length of hospital stay for patients with ICP was comparable to that reported in available literature [43]. In 13% of patients with RS, non-elective PICU admission was required, which was significantly higher than the 4% in the ICP group. Most commonly, in 72.7% of cases, PICU admission was required for reintubation. In the remaining patients that required PICU admission, admission was required for more intensive observation. Healthcare providers on the PICU have less patients per person, and therefore can monitor the patients better. Literature on PICU admission after palatoplasty is heterogeneous. Some studies have described PICU admission after every palatoplasty for patients with RS [16], whereas other articles did not provide data on postoperative PICU admission [19, 20, 28].

In this study, 38 syndromes were identified in the RS group. Stickler syndrome was the most prevalent syndrome, followed by Cornelia de Lange syndrome, 22q11-deletion syndrome, Nager syndrome, and Moebius syndrome. All of these syndromes are often associated with RS [10, 19, 44, 45].

In 2019, Bergeron et al. reported that patients with syndromic RS were more likely to have increased obstructive sleep apnea (OSA) than their non-syndromic RS counterparts [46]. They found no association between the baseline and postoperative OSA. Similarly, we found no association between the early PSG results and respiratory complications after palatoplasty. Postoperative respiratory problems often resolve within a few days and are, therefore, most likely explained by lingual and palatal swelling, which peaks 48 h after surgery [21, 32]. We speculate that preoperative weight, more specifically growth curves, is associated with the oropharyngeal luminal size, and more importantly, with general well-being; therefore, both might be associated with surgical outcomes. Delaying surgery for several weeks or additional postoperative surveillance in children with a low weight could alleviate the complications associated with a low preoperative weight.

This study has several limitations. First, this study had a retrospective design. Second, we were unable to assess the value of certain treatment modalities because the sample size was small. Third, we found that there were many missing data in the older electronic data files; therefore, the power of our results decreased slightly. Fourth, although the present study is the largest of its kind, the absolute sizes of the subgroups were small, which should be considered when interpreting the results. Lastly, we lacked an objective analysis of pre- and postoperative respiratory status.

Further studies are required to establish a better understanding of surgical outcomes in patients with RS and to determine the parameters that are crucial for surgical outcomes. Future research should include the creation of a large prospective database to further analyze the risk factors associated with surgical outcomes. We believe that identifying more accurate risk factors could drastically improve surgical outcomes in patients with RS in both high- and low-resource settings. However, to do so, a large amount of data is required, which can be acquired in a multicenter research study.

## Conclusion

The aim of this study was to compare, surgical outcomes after palatoplasty in patients with Robin sequence and isolated cleft palate. We have found that the incidence of respiratory complications is higher in the Robin sequence population. We found that patients with a lower preoperative weight, and patients with prior treatment with TLA had more respiratory complications and PICU admission. We conclude that closer postoperative surveillance should be considered in patients with these risk factors. In addition, this study raises questions about the optimal method to address primary respiratory problems in infants with severe Robin sequence after they are born and highlights the potential implications of these treatment choices.
